# Chemical Pressure‐Induced FWHM Narrowing in Narrowband Green Phosphors for Laser Displays with Ultra‐High Saturation Thresholds

**DOI:** 10.1002/advs.202505385

**Published:** 2025-06-04

**Authors:** Runtian Kang, Zhezhe Su, Chunxu Bao, Yuhua Wang

**Affiliations:** ^1^ National & local Joint Engineering Laboratory for Optical Conversion Materials and Technology School of Materials and Energy Lanzhou University Lanzhou 730000 China

**Keywords:** chemical pressure, high‐pressure, laser display, narrow‐band phosphor, spectral regulation

## Abstract

Due to its outstanding performance, laser display technology has received extensive attention. Specifically, in this study, a novel narrow‐band green phosphor, Na_0.6_Rb_0.4_BaB_9_O_15_ (NRBBO): Eu^2+^, for laser display is obtained. Under chemical pressure, Eu^2+^ migrates from Ba^2+^ to Na^+^ lattice sites in NRBBO, leading to a gradual decrease in the FWHM, making it more competitive for display applications. The existence of chemical pressure in the lattice is demonstrated through a high‐pressure spectrum. The migration of Eu^2+^ lattice sites under chemical pressure is verified by XRD refinement, low‐temperature spectrum and EXAFS. NRBBO: 0.11Eu^2+^ can be effectively excited by NUV and blue light, with an emission peak at 512 nm and a FWHM of 54 nm. It retains 82% of its luminescence intensity at 150 °C compared to room temperature. The luminous flux of NRBBO: 0.11Eu^2+^ under 90.7 W mm^−2^ laser excitation reaches 2081.6 lm without light saturation. Meanwhile, the excellent CL performance demonstrates that NRBBO: 0.11Eu^2+^ exhibits stability under high energy density. NRBBO: 0.11 Eu^2+^ – based WLED achieves a color gamut of 106% NTSC. The excellent performance of NRBBO: 0.11Eu^2+^ demonstrates great potential as a green phosphor for laser display applications.

## Graphical abstract

Spectral tailoring is achieved in the phosphor Na_0.6_Rb_0.4_BaB_9_O_15_ (NRBBO): Eu^2+^ using chemical pressure. The gradual decrease of FWHM under chemical pressure of Rb^+^ ions. WLED based on NRBBO: 0.11Eu^2+^ have a color gamut of 106% NTSC. The excellent laser excitation properties of NRBBO: 0.11Eu^2+^ give it great potential as a green phosphor for laser display.

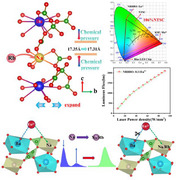

## 1 Introduction

As the most critical information output terminal in human‐computer interaction, displays have permeated every aspect of daily life. In the current era, a multitude of display technologies has continuously emerged. However, existing display light sources exhibit broad spectra. Due to excessively wide spectral widths, the three primary color spectra overlap during color mixing, making them indistinguishable to the human eye and leading to suboptimal display performance.^[^ ^1^, ^2^, ^3^, ^4^, ^5^, ^6^, ^7^, ^8^, ^9^ ^]^ With its ultra‐narrow emission bandwidth, laser displays enable ultra‐high color gamut and have increasingly gained traction in the field.^[^ ^10^, ^11^, ^12^, ^13^, ^14^ ^]^

The most prominent characteristic of laser display is that the laser spectrum is a line spectrum with a spectral width of less than 5 nanometers, which enables a large color gamut. This endows it with an unrivaled color reproduction ability. Moreover, laser display employs reflective imaging, which shares the same principle as the reflective light imaging of natural objects entering the human eye. The light is reflected to the human eye by the screen, and it is soft and not glaring, thereby conferring a high level of viewing comfort.^[^ ^15^, ^16^, ^17^ ^]^

Laser display light sources can be classified into three main types: three primary‐color pure laser sources, laser phosphor sources, and laser hybrid sources. The pure laser source directly uses red, green, and blue lasers as emitters.^[^ ^18^, ^19^ ^]^ The laser phosphor source employs a blue laser as the primary excitation source, generating red and green light via phosphor conversion. The laser hybrid source uses a blue laser for blue light, with red light generated by LEDs or lasers and green light still produced via a phosphor. Three‐primary‐color pure laser sources remain scarce due to green lasers' lower conversion efficiency and technical challenges. Manufacturing green laser diodes (LDs) with sufficient power for displays is significantly more difficult than for blue and red LDs. Additionally, increasing red LD power exacerbates thermal management challenges.^[^ ^20^, ^21^ ^]^ Consequently, most manufacturers prioritize improving monochromatic or bicolor source performance over developing three‐primary‐color pure lasers.

Consequently, phosphor‐converted laser display remains the dominant technology in current laser display applications.^[^ ^21^, ^22^ ^]^ The laser phosphor technology employs a single blue laser source to excite a rotating multicolor phosphor color wheel, effectively addressing thermal quenching and thermal management challenges. This allows the phosphor to operate stably under high‐intensity excitation. The rotating phosphor color wheel sequentially produces different colors, ultimately producing white light. This technology has overcome key technical challenges in efficiency and reliability, endowing laser displays with advantages including pure color, long lifespan, high safety, eye protection, and no risk of explosion or fragmentation.

In the context of ultra‐high color gamut fluorescence‐converting laser display applications, luminescent materials form the core of laser phosphor display technology. This, in turn, imposes greater requirements on red and green luminescent materials. To enhance the color gamut of display devices, researchers have made progress in exploring various narrow‐band light‐emitting materials. For instance, CsPbX_3_ (X = Cl, Br, I) possesses a full width at half‐maximum (FWHM) of less than 40 nm.^[^ ^23^, ^24^ ^]^ Nevertheless, the environmental instability of these materials restricts their commercial adoption. Concerning inorganic phosphors, commonly used ones in laser displays such as Y_3_Al_5_O_12_ (YAG): Ce^3+^ and CaAlSiN_3_: Eu^2+^ are limited by their wide FWHM, which prevents achieving ultra‐high color gamut displays.^[^ ^25^, ^26^ ^]^ Additionally, the synthesis conditions for the narrow‐band green phosphor β‐SiAlON: Eu^2+^ are quite demanding.^[^ ^27^ ^]^ The narrow‐band red phosphor K_2_SiF_6_: Mn^4+^ has a long decay time, and its preparation requires highly toxic HF, which has hindered its further application in laser displays.^[^ ^28^, ^29^ ^]^ Moreover, the intense thermal shock from laser light makes luminescent materials with lower physical and chemical stability unsuitable for laser display applications. Presently, most luminescent materials experience photothermal saturation. Specifically, β‐SiAlON: Eu^2+^ has a saturation power density of 22 W mm^−2^, YAG: Ce^3+^ of 10.5 W mm^−2^, and Mg_2_Al_4_Si_5_O_18_: Eu^2+^ glass of 3.25 W mm^−2^.^[^ ^25^, ^30^, ^31^ ^]^ These relatively low power densities present significant challenges to their practical implementation in laser display systems. Given the peak sensitivity of the human eye in the green spectral region, the development of novel narrow‐band green phosphors with high efficiency and high saturation thresholds plays a critical role in advancing next‐generation laser display technology.

Achieving narrow‐band green emission from Eu^2+^‐activated phosphors is typically challenging due to stringent requirements: a highly condensed crystal structure, a single luminescent center, and a symmetric coordination environment. In this manuscript, we investigated the narrow‐band green emission properties of NRBBO: Eu^2+^. The lamellar crystal structure of NRBBO exhibits a condensation degree of 𝜅 = 0.6 (B: O = 0.6), ensuring a well‐condensed crystalline framework. Additionally, the highly symmetric [NaO_6_] polyhedra within the NaBaB_9_O_15_ (NBBO) structure facilitates the generation of narrow‐band emission from Eu^2+^ ions. However, there are two luminescent centers Ba^2+^ and Na^+^ in NBBO, which makes it difficult for Eu^2+^ to produce narrow‐band luminescence in NBBO. Therefore, how to make Eu^2+^ occupy the lattice site of Na^+^ in NBBO to achieve narrow‐band emission has important research value. In this paper, we achieved the tailoring and narrowing of the emission spectrum through the chemical pressure exerted by Rb^+^ on Na^+^ in NBBO: Eu^2+^. The influence mechanism of chemical pressure on spectral tailoring and narrowing was discussed in detail. In addition, NRBBO: Eu^2+^ has excellent application value in laser display. More importantly, our research clearly shows that using chemical pressure to regulate the switching of activator ions between different lattice sites is an effective strategy for obtaining narrow‐band emitting phosphors, which will guide researchers to design other new types of narrow‐band phosphors.

## 2 Results and Discussion

### 2.1 Crystal Structure and Morphology

**Figure 1:**
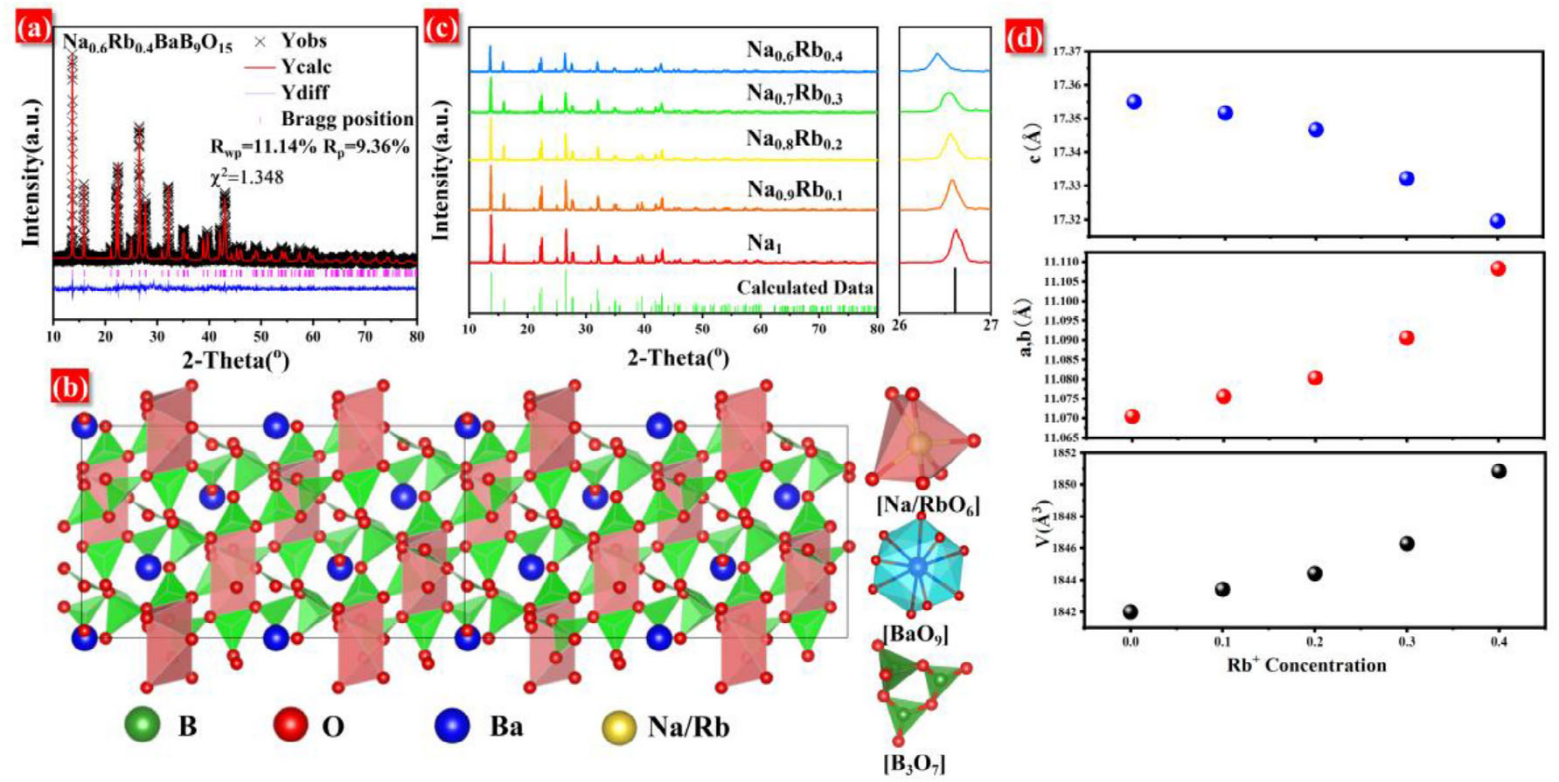
a) Refined XRD pattern of the NRBBO sample. b) Schematic illustration of the crystal structure of the NRBBO matrix, including the coordination polyhedra of Na and Ba. c) XRD patterns of the N_1‐x_R_x_BBO: Eu^2+^ (0 ≤ x ≤ 0.4) series of samples. d) Variation of lattice parameters and unit cell volumes for the N_1‐x_R_x_BBO: Eu^2+^ (0 ≤ x ≤ 0.4) series of samples.

The XRD of NRBBO was refined using the structural data of NaBaB_9_O_15_ reported by Penin et al., as presented in **Figure** **1**a.^[^ ^32^ ^]^ The experimental and calculated data match closely, with low residual factors (χ^2^ = 1.348, R_p_ = 9.36%, R_wp_ = 11.14%), indicating that the refinement has converged well. The crystallographic data of the NRBBO are listed in **Table** **1**, other crystallographic data for samples with different Rb^+^ concentrations are presented in Tables S1–S4 (Supporting Information). Figure 1b shows the crystal structure model of NRBBO, the crystal structure of NRBBO belongs to the trigonal system with the space group *R*3*c*. The structure features two planar [BO_3_] units and one [BO_4_] unit connected via shared oxygen vertices, forming a ring structure. The main framework of NRBBO comprises [B_3_O_7_] rings stacked along the [001] direction, creating one‐dimensional channels. Ba^2+^ ions are coordinated by nine oxygen atoms to form [BaO_9_] polyhedra, while Na⁺/Rb⁺ ions adopt six‐coordinate [Na/RbO_6_] octahedra. These cations alternate within the tunnels, thereby constructing the full NRBBO crystal structure. The dense packing of this framework underlies its excellent physicochemical stability.^[^ ^14^ ^]^

**Table 1:** Crystallographic data of Na_0.6_Rb_0.4_BaB_9_O_15_ by the Rietveld refinement.

	Na_0.6_Rb_0.4_BaB_9_O_15_
Space group	*R*3*c*
Crystal system	trigonal
Cell parameters (Å)	a = b = 11.1104(3) c = 17.3154(7)
Cell ratio	c/a = 1.5585
Z	6
Cell volume (Å^3^)	1851.06(10)
Reliability factors	χ^2^ = 1.348, R_p_ = 9.36%, R_wp_ = 11.14%

Figure 1c presents the X‐ray diffraction (XRD) patterns of the Na_1‐x_Rb_x_BaB_9_O_15_ (N_1‐x_R_x_BBO): Eu^2+^ (0 ≤ x ≤ 0.4). All diffraction peaks of the samples match well with calculated values, with no impurity phases observed, confirming their single‐phase nature. Notably, as the concentration of Rb^+^ increases, the diffraction peaks shift from higher to lower 2θ angles. This shift is attributed to the incorporation of Rb^+^ into the crystal lattice. The ionic radii of Rb^+^ and Na^+^ (with a coordination number of 6) are 1.52 and 1.02 Å, respectively. The larger radius of Rb^+^ leads to lattice expansion when it substitutes for the smaller Na^+^, resulting in the observed decrease in 2θ angles. To further verify that the shift of the diffraction peaks is caused by the lattice expansion due to the introduction of Rb^+^, the detailed lattice parameters of the series of N_1‐x_R_x_BBO: Eu^2+^ (0 ≤ x ≤ 0.4) samples were obtained by Rietveld refinement, as shown in Figure 1d. It is obvious from Figure 1d that the cell volume expands with increasing Rb^+^ concentration, which is consistent with the analysis above. This reinforces the fact that Rb^+^ successfully enters the NBBO lattice and replaces the Na^+^ lattice site.

**Figure 2:**
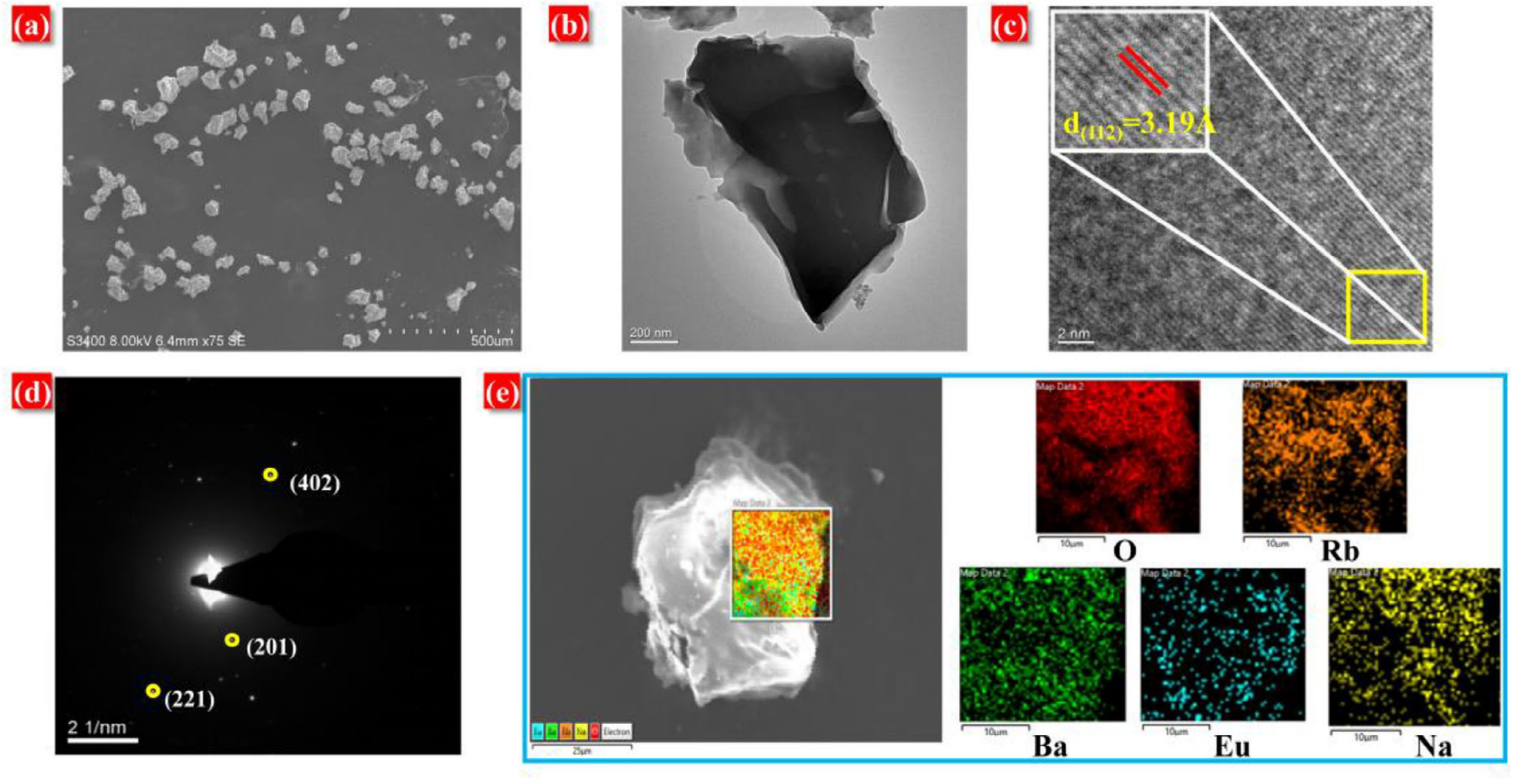
a) The SEM image of NRBBO: Eu^2+^. b) The TEM image of NRBBO: Eu^2+^. c) The HRTEM image of NRBBO: Eu^2+^. d) The SAED image of NRBBO: Eu^2+^. e) EDX mapping of NRBBO: Eu^2+^.

Morphological characterization of NRBBO: Eu^2+^ was performed via scanning electron microscopy (SEM) and transmission electron microscopy (TEM), as shown in **Figure** **2**a,b. The SEM image in Figure 2a reveals that the NRBBO: Eu^2+^ sample exhibits an irregular morphology with good dispersion. TEM imaging (Figure 2b) indicates smooth surface morphology, suggesting strong crystallinity. Figure 2c presents the high‐resolution TEM (HRTEM) image of NRBBO Eu^2+^, where distinct lattice fringes are observed. This reflects that the sample has good stability under high‐energy electron bombardment. The calculated distance between two adjacent crystal faces is ≈3.19 Å, which corresponds to the crystal face of (112) in the NRBBO: Eu^2+^. Figure 2d presents a selected area electron diffraction (SAED) image of an NRBBO: Eu^2+^, where distinct diffraction spots are observable. The labeled spots (221), (402), and (201) further confirm the well‐crystallized nature of the NRBBO: Eu^2+^ sample. Energy‐dispersive x‐ray spectroscopy (EDS) analysis (Figure 2e) confirms the presence of Na, Ba, Rb, O, and Eu in the sample, with no impurity elements detected. Homogeneous distribution of these elements within NRBBO: Eu^2+^ further validates successful synthesis.

### 2.2 Luminescence Performance

**Figure 3:**
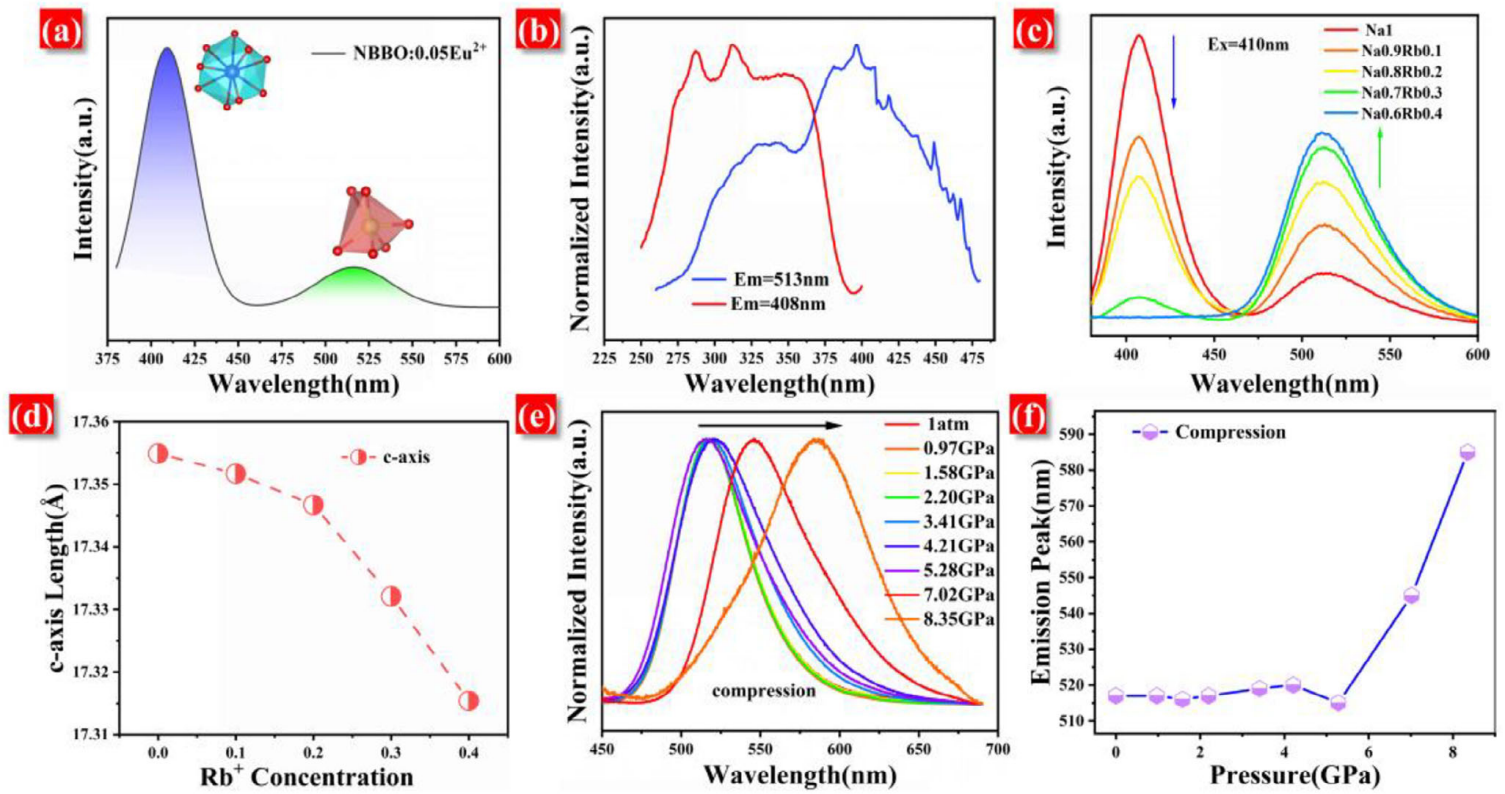
a) Emission spectrum of NBBO: 0.05Eu^2+^. b) Excitation spectra of NBBO: 0.05Eu^2+^ at 408 and 513 nm detection. c) Emission spectra of N_1‐x_R_x_BBO: 0.05Eu^2+^ (0 ≤ x ≤ 0.4) series samples. d) Variation of c‐axis length with Rb^+^ doping concentration. e) The emission spectra of NRBBO: 0.11Eu^2+^ under different pressures. f) The emission peak of NRBBO: 0.11Eu^2+^ varies with pressure.

**Figure** **3**a presents the emission spectrum of the NBBO: 0.05Eu^2+^, which features two distinct peaks centered at 408 nm (ultraviolet region) and 513 nm (green region). To investigate the origins of these luminescent centers, excitation spectra associated with each emission band were analyzed, as shown in Figure 3b. The results reveal distinct excitation spectral profiles for the two emission bands. Specifically, the emission peak at 408 nm is excited by ultraviolet (UV) covering 250–375 nm, while the emission peak at 513 nm corresponds to the excitation spectrum extending from near‐ultraviolet (NUV) to the blue region (300‐475 nm). This differentiation confirms that the two emission peaks originate from Eu^2+^ occupying distinct sites within the crystal lattice. According to the Hume‐Rothery rule, the difference in ionic radii between the dopant and the host cation should not exceed 30%.^[^ ^33^ ^]^ Using Calculation S1 (Supporting Information), we can calculate the radius difference of the ions. Ba^2+^ has a radius of 1.47 Å (CN = 9), Eu^2+^ has radii of 1.30 Å (CN = 9) and 1.17 Å (CN = 6), and Na^+^ has a radius of 1.02 Å (CN = 6). The calculated Dr_1_ value for the radii of Ba^2+^ and Eu^2+^ in the [BaO_9_] polyhedron is 11.56%, while the Dr_2_ value for Na^+^ and Eu^2+^ in the [NaO_6_] polyhedron is 14.71%. Since Dr_2_ exceeds Dr_1_, and considering the significant valence mismatch between Eu^2+^ and Na^+^, which inhibits the occupation of Na^+^ sites by Eu^2+^, it follows that Eu^2+^ preferentially occupies the Ba^2+^ sites within the NBBO structure.

Figure 3a illustrates that the emission peak at 408 nm is significantly more intense than that at 513 nm. This suggests that the majority of Eu^2+^ ions preferentially occupy the Ba^2+^ lattice sites in substantial quantities, leading to the predominant UV emission within the NBBO system. Conversely, a smaller fraction of Eu^2+^ ions occupy the Na^+^ lattice sites, resulting in the observed green emission. As a result, the NBBO: 0.05Eu^2+^ exhibits bimodal emission, characterized by a much stronger intensity of UV emission compared to green emission. To further support this observation, we can analyze the luminescence centers using Calculation S2 (Supporting Information).^[^ ^34^ ^]^ In Calculation S2 (Supporting Information), Ea and V are constants specific to the phosphor system, indicating that the energy (E) of the emission peak is primarily influenced by the values of r and n. Specifically, a larger value of nr correlates with a higher energy E and a shorter emission wavelength. Since Eu^2+^ occupies the Ba^2+^ lattice sites within the [BaO_9_] polyhedra, it results in a larger nr value, thus producing shorter wavelength emissions. Therefore, we can conclude that the emission peak at 408 nm is attributed to Eu^2+^ at the Ba^2+^ lattice sites, while the emission peak at 513 nm arises from Eu^2+^ at the Na^+^ lattice sites.

Figure 3c presents the emission spectra of the N_1‐x_R_x_BBO: 0.05Eu^2+^ (0 ≤ x ≤ 0.4). As illustrated, the emission peaks at 408nm decreases with increasing Rb^+^ concentration, while the intensity of the green emission peaks correspondingly increases. Notably, at an Rb^+^ concentration of 0.4, the emission peak at 408 nm vanishes completely, and the intensity of the green emission peak reaches its maximum. The FWHM of NRBBO: Eu^2+^ obtained at this time was only 54 nm. This spectral clipping phenomenon warrants further investigation into the crystal structure of the N_1‐x_R_x_BBO: Eu^2+^. Figure 3d shows the variation of the c‐axis length with the concentration of Rb^+^. It has been previously determined that Rb^+^ occupies the Na^+^ lattice sites in the NRBBO structure. Figure 3d indicates that the c‐axis length gradually decreases as the Rb^+^ concentration increases. This shrinkage of the c‐axis is attributed to the chemical pressure exerted by Rb^+^. The nature of this chemical pressure can be interpreted as an elastic lattice strain, which can be quantitatively described using Equation (1).^[^ ^35^ ^]^ This equation allows for the assessment of the impact of Rb^+^ doping on the overall lattice structure and the effects on the emission properties of the Eu^2+^ within the phosphor matrix. By understanding these structural changes, we can better elucidate the mechanisms behind spectral clipping.

(1) $$d_{p}=-E\frac{d_{x}}{x_{0}}$$

where d_p_ is the chemical pressure, d_x_ is the change in c‐axis length, x_0_ is the initial c‐axis length, and E is Young's modulus. In the NRBBO system, the values of E and x_0_ are fixed, meaning that the chemical pressure (d_p_) is solely dependent on the change in d_x_. The length of the c‐axis gradually decreases as the concentration of Rb^+^ increases. According to Equation (1), it can be shown that as the doping concentration of Rb^+^ increases, the positive chemical pressure exerted by Rb^+^ on the c‐axis also intensifies. To confirm that Rb^+^ doping generates chemical pressure within the lattice, we conducted high‐pressure spectral tests on NRBBO: 0.11Eu^2+^, as shown in Figure 3e. Typically, when phosphors are subjected to external pressure, the lattice contracts, resulting in a redshift in the emission spectrum.^[^ ^36^, ^37^ ^]^ Figure 3e shows the normalized emission spectrum of NRBBO: 0.11Eu^2+^. Figure 3f shows the curve of the peak position of the emission spectrum of NRBBO: 0.11Eu^2+^ varying with pressure. It can be seen from Figure 3f that the peak of the emission spectrum of NRBBO: 0.11Eu^2+^ remains unchanged in the pressure range from 0 to 5.12 GPa. However, after 5.12 GPa, a significant red‐shift occurs in the emission spectrum. Specifically, under the isostatic pressure of 8.35 GPa, the emission peak shifts to 585 nm. This shift can be attributed to the chemical pressure within the lattice induced by Rb^+^ doping. As external isostatic pressure increases, it gradually counteracts the internal chemical pressure generated by the Rb^+^ ions. Once the external pressure surpasses 5.12 GPa, it exceeds the internal chemical pressure, resulting in the observed redshift in the emission spectrum. This analysis of the high‐pressure spectra from the NRBBO: 0.11Eu^2+^ confirms that Rb^+^ doping indeed creates chemical pressure within the lattice. In addition, it can be seen from Figure 3f that when the pressure increases from 5.12 to 8.35 GPa, the emission peak position of NRBBO: 0.11Eu^2+^ shifts from the green light at 520 nm to the yellow light region at 585 nm. Under a pressure difference of 3.23 GPa, a huge redshift of 65 nm is produced. Meanwhile, green and yellow lights are extremely sensitive to the human eye. Therefore, when NRBBO: 0.11Eu^2+^ is used as a pressure alarm, it has a good visual advantage.

**Figure 4:**
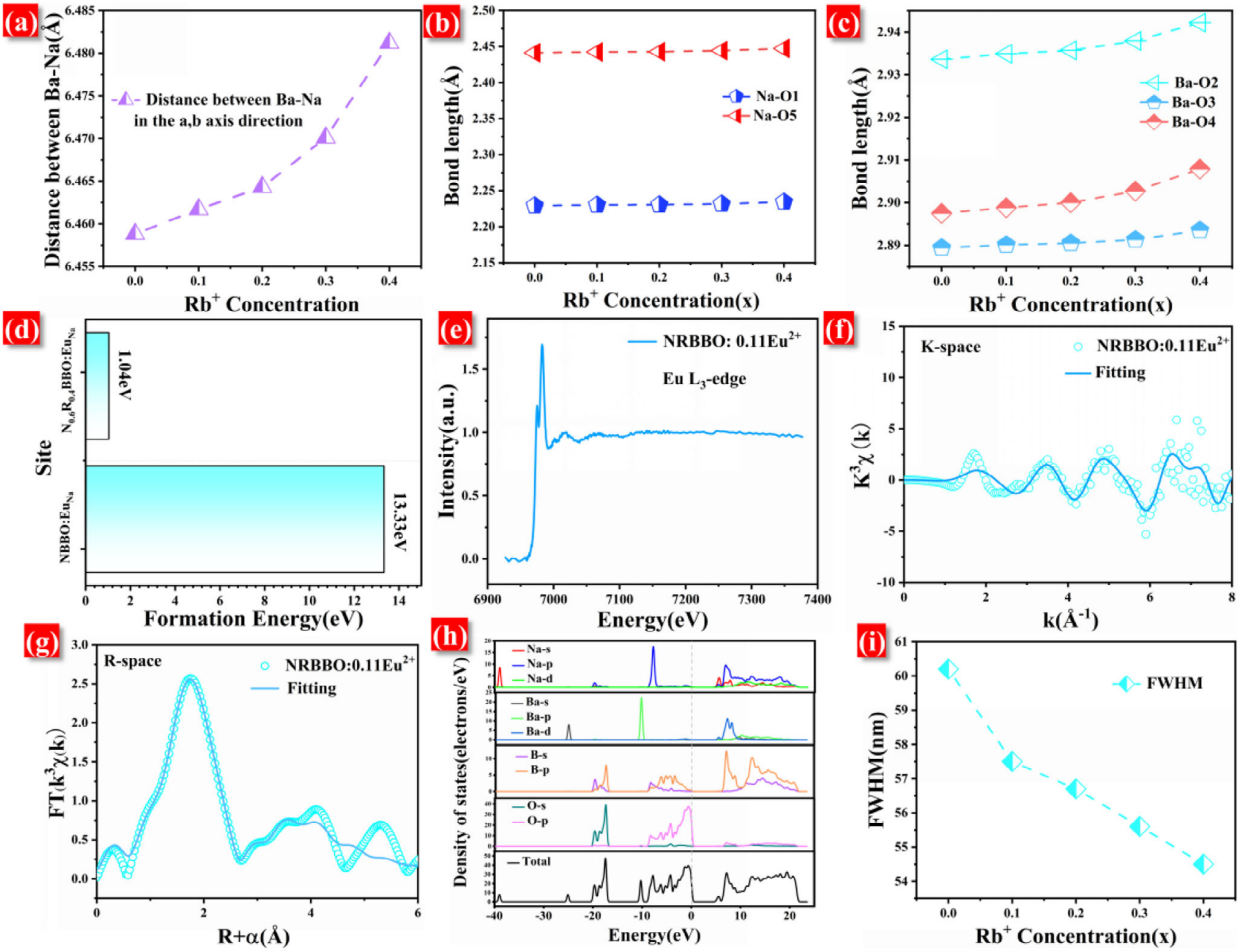
a) Variation of Ba‐Na distance with Rb^+^ concentration in the a, b‐axis direction. b) Variation of [NaO_6_] polyhedra Na─O bond length with Rb^+^ doping concentration. c) Variation of [BaO_9_] polyhedra Ba‐O bond length with Rb^+^ doping concentration. d) The formation energies of Eu^2+^ substituting for Na sites in NBBO and NRBBO. e) The Eu L3‐edge EXAFS spectrum of NRBBO: 0.11Eu^2+^. f) The Eu L3‐edge k3‐weighted EXAFS for NRBBO: 0.11Eu^2+^ in k‐space. g) Fourier transformed‐extended X‐ray absorption fine structure fitting for NRBBO: 0.11Eu^2+^ in R‐space. h) The density of states of NRBBO. i)Variation of FWHM of green emission peak with Rb^+^ concentration for N_1‐x_R_x_BBO: Eu^2+^.

To understand the mechanism by which the chemical pressure from Rb^+^ affects the luminescence of NRBBO: Eu^2+^, we investigated the variations in the bond lengths of the [NaO_6_] and [BaO_9_] polyhedra with increasing Rb^+^ doping concentration. As previously noted, in the absence of Rb^+^ doping and chemical pressure, Eu^2+^ preferentially occupies the lattice sites of Ba^2+^ due to their similar valence states, leading to predominantly blue luminescence in the samples. With the introduction of Rb^+^, the chemical pressure exerted on NRBBO in the c‐axis direction increases gradually. This increase in chemical pressure causes elastic strain within the lattice, resulting in an expansion of the lattice in the a‐ and b‐axis directions. This expansion alters the local environment around the Eu^2+^. Consequently, the changes in bond lengths and lattice parameters due to Rb^+^ doping and the resulting chemical pressure can significantly influence the luminescent properties of the phosphors, potentially leading to shifts in emission color or intensity. As shown in **Figure** **4**a, the distance between Ba‐Na gradually increases with the increase of Rb^+^ concentration in the a, b‐axis direction, which also verifies that the lattice will expand in the a, b‐axis direction under the pressure in the c‐axis. Figure 4b,c illustrates that as the concentration of Rb^+^ increases, the Na/Rb─O and Ba─O bond lengths progressively lengthen. This elongation ultimately results in the expansion of the [Na/RbO_6_] and [BaO_9_] polyhedra. To maintain lattice stability amid this polyhedra expansion, Eu^2+^ ions gradually migrate towards the larger Rb^+^ lattice sites under chemical pressure, rather than remaining in the Ba^2+^ sites, which would exacerbate the expansion of the polyhedra. Additionally, Figure 4b,c reveal that the change in Ba─O bond length is more pronounced than that of the Na/Rb─O bond length. This discrepancy arises because the ionic radius of Eu^2+^ is smaller than that of Ba^2+^ and Rb^+^, but larger than that of Na^+^. Consequently, the alteration in Ba─O bonds becomes more evident as Eu^2+^ migrates from the Ba^2+^ sites to the Na/Rb lattice sites. This migration is further supported by the observed gradual transition of Eu^2+^ from the Ba lattice site to the Na/Rb sites.

**Figure 5:**
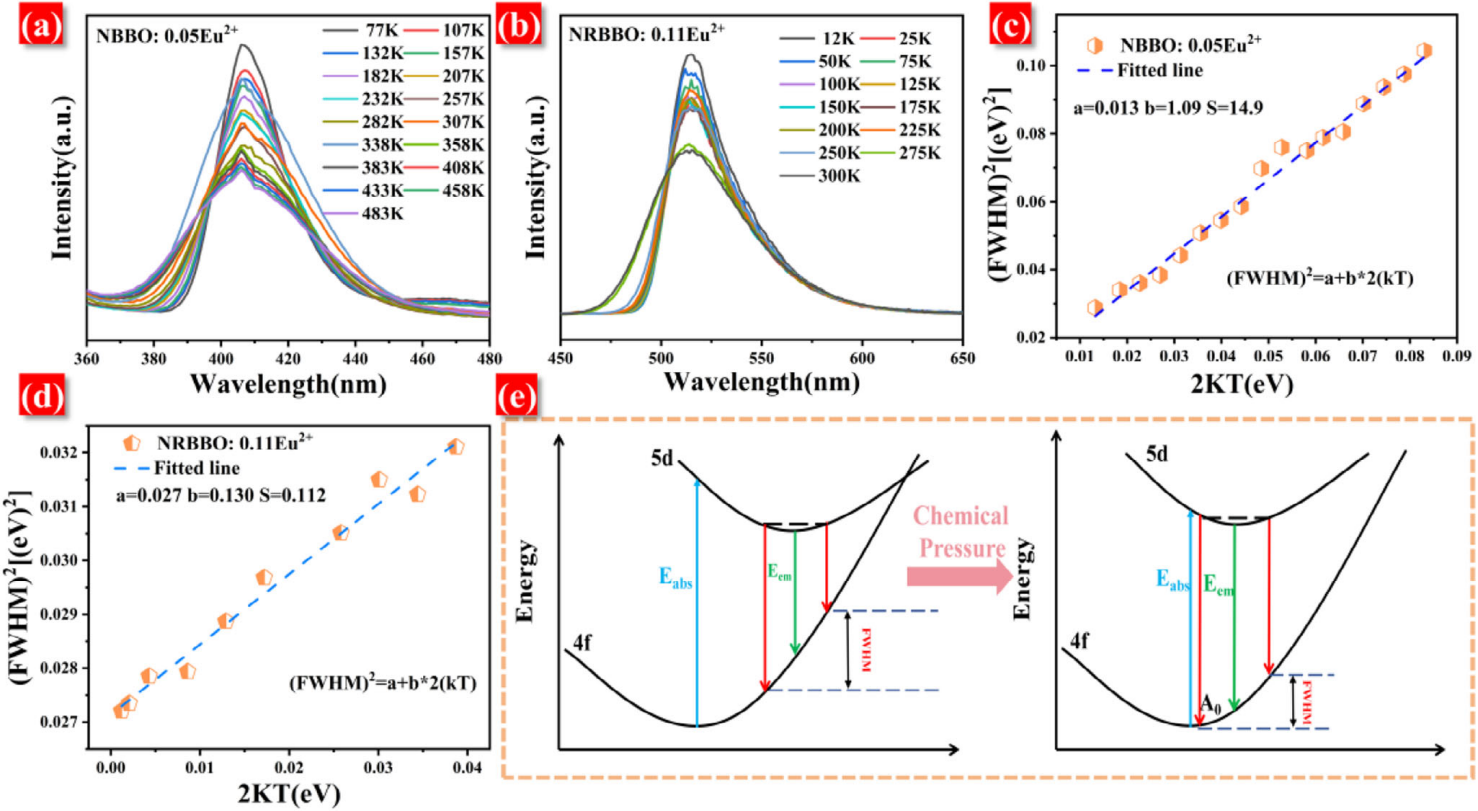
a)Variation of emission spectra of NBBO: 0.05Eu^2+^ with temperature. b) Variation of emission spectra of NRBBO: 0.11Eu^2+^ with temperature. c) Fitting results of (FWHM)^2^ as a function of 2kT for NBBO: 0.05Eu^2+^. d) Fitting results of (FWHM)^2^ as a function of 2kT for NRBBO: 0.11Eu^2+^. e) Schematic representation of the effect of structural relaxation on the FWHM of the emission spectrum of N_1‐x_R_x_BBO: Eu^2+^.

In addition, we also calculated the formation energies of Eu^2+^ substituting for the Na^+^ sites in NBBO and NRBBO, as shown in Figure 4d. The formation energies of Eu^2+^ substituting for the Na^+^ sites in NBBO and NRBBO are 13.33 and 1.04 eV respectively (Calculation S3, Supporting Information). The lower the doping formation energy, the easier the substitution. This indicates that with the doping of Rb^+^, Eu^2+^ will more easily occupy the Na^+^ sites. To further confirm that Eu^2+^ is indeed transitioning to occupy the [Na/RbO_6_] polyhedra under Rb^+^ chemical pressure, we analyzed the Eu L3‐edge extended x‐ray absorption fine structure (EXAFS) spectrum of the NRBBO: 0.11Eu^2+^ phosphor, as depicted in Figure 4e. EXAFS spectrum can provide information about the electronic and local geometry of specific elements in the structure. To further study the coordination environment of Eu^2+^ in the NRBBO: 0.11Eu^2+^ crystal, we performed a Fourier transform on the EXAFS curve. The Fourier‐transform fitted spectra are shown in **Figure** **5**f,g. This fitting allowed us to extract structural configuration information regarding the local atomic environment surrounding the Eu atoms through Fourier transform. The coordination shells of NRBBO: 0.11Eu^2+^ were fitted by the contributions of Na‐O1, Na‐O5, and Na‐B1 contributions fitted. The fact that the data can be fitted well suggests that Eu^2+^ would occupy the [Na/RbO_6_] polyhedra in NRBBO: 0.11Eu^2+^. This conclusion has proven that Eu^2+^ will enter the [Na/RbO_6_] polyhedra under the action of chemical pressure.^[^ ^38^, ^39^ ^]^

We carried out first‐principles calculations on NRBBO, and the energy‐band diagram is shown in Figure S21 (Supporting Information). The compound NRBBO is an indirect band‐gap material with a band‐gap width of 4.92 eV. The relatively large band gap can ensure that NRBBO is a suitable host for Eu^2+^. As shown in Figure 4h, the electronic states behind the Fermi energy are mainly composed of O p and a small amount of B p states. The bottom of the CB is mainly composed of a mixture of B p states, Ba d, and Na p states. Therefore, we conclude that the band‐gap transition of the compound NRBBO mainly occurs in the O p → B p transition and the states of the lowest forbidden band mainly come from the [B_3_O_7_] group. In contrast, the Na^+^ atoms contribute less to these states. Therefore, when Eu^2+^ ions are doped into the Na^+^ sites, the probability of generating free carriers under radiation is reduced, which is beneficial to the photoluminescence process. This is also the fundamental reason why NRBBO: Eu^2+^ has high luminous efficiency.

Finally, the spectral analysis presented in Figure 4i illustrates the variation in the FWHM of the green emission peak for N_1‐x_R_x_BBO: Eu^2+^ (0 ≤ x ≤ 0.4) as Rb^+^ concentration increases. The data show that as the concentration of Rb^+^ increases, the FWHM of the green emission peak gradually decreases. When the Rb^+^ concentration is 0.4, the FWHM decreases from the initial 60 to 54 nm.

The emission spectrum of Eu^2+^ doped phosphors is known to be narrowed by smaller lattice relaxations. To further explain the phenomenon of narrowing FWHM in N_1‐x_R_x_BBO: Eu^2+^, we tested the low‐temperature spectra of NBBO: Eu^2+^ and NRBBO: Eu^2+^, and the results are shown in Figure 5a,b. For Eu^2+^‐activated phosphors, lattice relaxation is closely related to the coupling of excited‐state electrons through lattice vibrations. Specifically, the smaller the coupling strength between the two, the stronger the lattice relaxation. The lattice coupling strength can be evaluated with the help of the Huang‐Rhys factor (S), and the larger the S value, the stronger the coupling strength. It is generally considered that S < 1 is a weak coupling, 1 < S < 5 is a moderate coupling, and S > 5 is a strong coupling.^[^ ^40^ ^]^ The value of S can be calculated according to Calculation S4 (Supporting Information).^[^ ^41^, ^42^, ^43^ ^]^ Figure 5c,d demonstrates the fitting of (FWHM)^2^ as a function of 2kT for the NBBO: Eu^2+^ and NRBBO: Eu^2+^. It is calculated that the S is 14.9 for the NBBO: Eu^2+^ and 0.112 for the NRBBO: Eu^2+^. This indicates that Rb^+^ doping reduces coupling between Eu^2+^ excited‐state electrons and lattice vibrations in N_1‐x_R_x_BBO: Eu^2+^. As shown in Figure 5e, Rb^+^ doping induces a gradual increase in chemical pressure, leading to reduced lattice relaxation and a correspondingly narrower emission FWHM.

**Figure 6:**
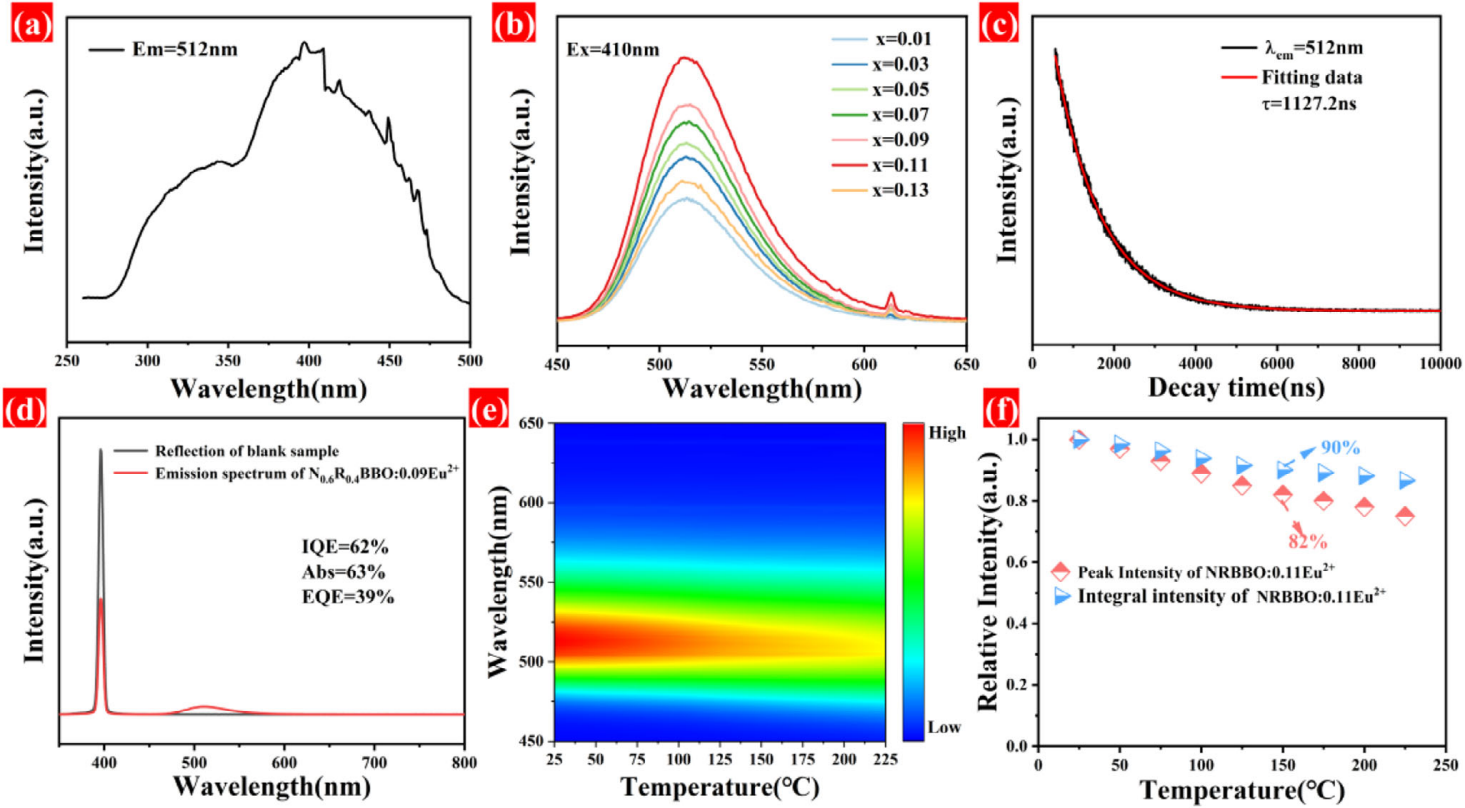
a) Excitation spectrum of NRBBO: 0.11Eu^2+^. b) Emission spectra of NRBBO: xEu^2+^ (0.01 ≤ x ≤ 0.13). c) The fluorescence decay curve of NRBBO: 0.11Eu^2+^. d) The quantum efficiency spectrum of NRBBO: 0.11Eu^2+^. e) 2D plot of the temperature‐dependent emission spectra of NRBBO: 0.11Eu^2+^. f) Variation of the normalized peak emission intensity and integral emission intensity of NRBBO: 0.11Eu^2+^ with temperature.

**Figure** **6**a shows the excitation spectrum of NRBBO: 0.11Eu^2+^. It exhibits broadband excitation, effectively covering the NUV to the blue region. This wide excitation ensures that it is efficiently excited by blue laser, making it suitable for laser display applications. The emission spectra of NRBBO: xEu^2+^ (0 ≤ x ≤ 0.13) samples were analyzed to determine the optimal doping concentration of Eu^2+^ in NRBBO, as illustrated in Figure 6b. Under 410 nm excitation, the luminescence intensity is first enhanced and then weakened with the increase of Eu^2+^ concentration, and the emission is the strongest when the doping concentration of Eu^2+^ is 0.11. After that, the luminous intensity gradually decayed due to the concentration quenching. The emission spectrum of the sample reveals a distinct peak at 620 nm corresponding to the emission of Eu^3+^. To reduce the concentration of Eu^3+^, the sample underwent heat treatment, and the resulting emission spectrum is illustrated in Figure S3 (Supporting Information). Notably, the luminous intensity of the heat‐treated sample is significantly enhanced, with the emission peak of Eu^3+^ disappearing entirely, as shown in the same figure. The luminescence intensity of the heat‐treated sample is three times that of the untreated sample. This increase in luminescence intensity following heat treatment can be attributed to the further reduction of Eu^3+^ ions and a corresponding increase in the concentration of Eu^2+^. As shown in Figure 6c, the decay time of NRBBO: 0.11Eu^2+^ is 1127.2 ns. The shorter decay time ensures that it is efficiently excited by the blue laser, which ensures that it can be used in laser display applications. Additionally, the quantum efficiency of NRBBO: 0.11Eu^2+^ was evaluated, as presented in Figure 6d. The internal quantum efficiency was found to be 61%, while the external quantum efficiency reached 39%. The high quantum efficiency values indicate promising potential for applications in laser displays.

Display devices often face challenges related to heat generation during prolonged use. If the luminescent materials used in these devices degrade significantly at high temperatures, it can lead to color drift, adversely affecting the visual quality and overall viewing experience. This issue is particularly pronounced in laser displays, which have high power densities that concentrate heat, increasing the risk of thermal degradation in luminescent materials.^[^ ^44^, ^45^ ^]^ Therefore, assessing the thermal stability of these materials is crucial for evaluating their performance. To evaluate the thermal stability of NRBBO: 0.11Eu^2+^, temperature‐dependent emission spectra were recorded. Figure 6e presents the 2D plot of the temperature‐dependent emission spectra of NRBBO: 0.11Eu^2+^. The results indicate that the sample exhibits excellent thermal stability. Specifically, as shown in Figure 6f, the peak and integral emission intensities of the NRBBO: 0.11Eu^2+^ at 150 °C maintain 82% and 90%, respectively, of those maintained at room temperature. This demonstrates the material's excellent resistance to thermal quenching, highlighting its potential as a promising candidate for high‐temperature laser display systems.

## 3 Application

### 3.1 Laser Properties

High‐power lasers, particularly blue laser with high power density, pose significant challenges when they irradiate phosphors. The extreme thermal shock from such lasers can lead to thermal saturation of the phosphor, which is a critical barrier to achieving high brightness and luminous flux in laser displays.^[^ ^46^ ^]^ One of the mechanisms of thermal saturation is the thermal quenching of the phosphor, which is closely related to the heat generation, heat dissipation, and heat resistance of the phosphor. To mitigate thermal saturation, enhancing heat dissipation is essential.^[^ ^47^ ^]^ Many researchers have focused on developing new materials that exhibit efficient and thermally stable luminescence, including single‐crystal phosphors, polycrystalline ceramic phosphors, and phosphors embedded in glass (PiG). Within the same phosphor material, improving heat dissipation can be accomplished through various strategies, such as increasing the thermal conductivity of the phosphor or implementing effective thermal management techniques, like metal heat sinks. However, the concentrated area of the incident laser spot will cause rapid heat accumulation, ultimately leading to a rapid rise in the temperature of this local area. This can result in thermal bursts or, in extreme cases, a complete luminescence failure. In contrast, using a high‐speed rotating fluorescent color wheel can significantly alleviate this issue. The rapid rotation of the wheel prevents heat from accumulating on the phosphor, thereby enhancing heat dissipation and raising the thermal saturation threshold of the laser‐excited phosphor. In this study, we evaluated the laser display performance of the NRBBO: 0.11Eu^2+^ using a high‐speed rotating phosphor wheel test system.

**Figure 7:**
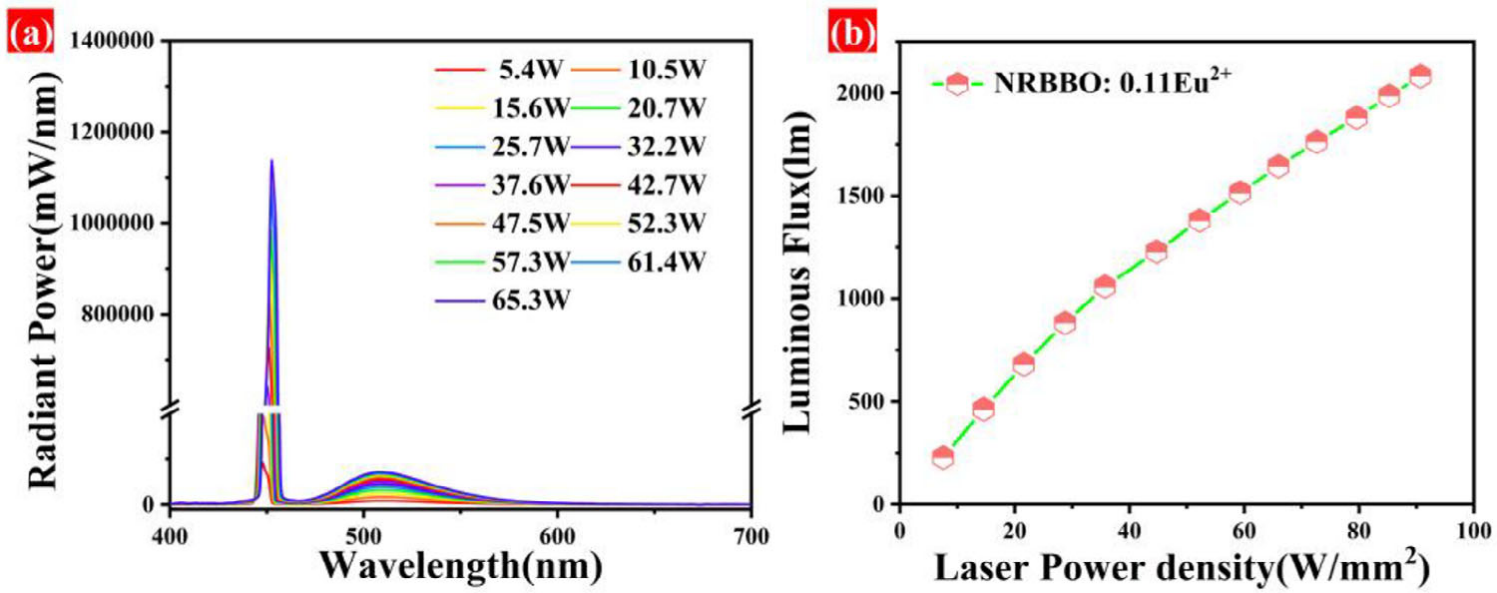
a) Emission spectra of NRBBO: 0.11Eu^2+^ under different laser power excitation. b) Variation of the luminous flux of NRBBO: 0.11Eu^2+^ with incident laser power density.

As shown in the curves in **Figure** **7**a,b, the emission intensity of the NRBBO: 0.11Eu^2+^ increases monotonically with the increase of the laser power density from 7.5 to 90.7 W mm^−2^, and no luminescence saturation occurs, which is seldom reported for many lasers driven phosphors. The luminous flux of the NRBBO: 0.11Eu^2+^ is 2081.2 lm at a power density of 90.7 W/mm^2^, which indicates that the NRBBO: 0.11Eu^2+^ is sufficiently stable for applications in laser displays. We also evaluated the performance of NRBBO: 0.11Eu^2+^ against previously reported green luminescent materials under laser excitation, as summarized in **Table** **2**. The comparison indicates that NRBBO: 0.11Eu^2+^ has an ultra‐high saturation threshold, which significantly exceeds that of other mainstream materials reported currently. Moreover, its luminous flux is also obviously superior to that of other materials. These findings have proven the excellent laser excitation characteristics of NRBBO: 0.11Eu^2+^, highlighting its great potential as a green component in laser display technology.

**Table 2:** Comparison of the laser excitation performance of NRBBO: 0.11Eu^2+^ with other phosphors reported.

Phosphor	Luminous flux [lm]	Saturation threshold [W mm^−2^]	
NRBBO: 0.11Eu^2+^	2081.2	90.7	This work
β – SiAlON: Eu^2+^	1310 lm	22	[30]
Y_3_Al_5_O_12_: Ce^3+^	1350	10.5	[48]
Lu_3_Al_5_O_12_: Ce^3+^ PiG	828.28	5.25	[49]
Gd_3_Al_5_O_12_: Ce^3+^ PiG	81.4	0.634	[50]
CaAlSiN3: Eu^2+^ PiG	49.3	1.9	[26]
Mg_2_Al_4_Si_5_O_18_: Eu^2+^ glass	273	3.25	[31]

### 3.2 Cathodoluminescence (CL) Properties

The luminescence of phosphors under cathode‐ray excitation requires the phosphors to work under the bombardment of an electron beam with high energy density. This is similar to the situation where phosphors under laser excitation need to be exposed to a laser with high energy density. Therefore, to evaluate the tolerance of NRBBO: 0.11Eu^2+^ when working under high energy, we investigated the cathodoluminescence (CL) properties of NRBBO: 0.11Eu^2+^. As shown in Figure S4a (Supporting Information), NRBBO: 0.11Eu^2+^ can produce green emission light with a peak at 512 nm under electron beam excitation. To further investigate the effects of current and voltage on CL performance, we tested the CL characteristics of NRBBO: 0.11Eu^2+^ under different voltage and current conditions. Figure S4a (Supporting Information) illustrates the relationship between CL intensity and voltage when the detection current is fixed at 40 mA. As the voltage varies within the range of 6–10 kV, the CL intensity increased linearly and no voltage saturation was observed, while the spectral shape and peak position remained stable. Figure S4b (Supporting Information) shows the relationship between different probe currents and CL intensity when the voltage is fixed at 6 kV. When the current increases from 40 to 80 mA, the CL intensity is significantly enhanced, indicating that NRBBO: 0.11Eu^2+^ exhibits excellent CL performance. Under the condition of a detection current of 60 mA, as the voltage increases, the penetration depth also increases, leading to an enhancement in cathodoluminescence intensity. The penetration depth (L) can be obtained through Calculation S5 (supporting information).^[^ ^51^ ^]^ When the voltage is 6, 7, 8, 9, and 10 kV, the penetration depths are 1052.22, 1927.26, 3255.49, 5169.42, and 7817.83 nm, respectively. The phenomenon of CL in phosphors is realized through the mechanism of secondary electron excitation. As the electron penetration depth increases, the number of secondary electrons generated continuously increases, which effectively excites more Eu^2+^ ions, ultimately leading to a significant enhancement in CL intensity.

For cathode ray luminescent materials, it is crucial to withstand long‐term bombardment by electron beams without decomposition. To study the stability of NRBBO: 0.11Eu^2+^ under electron beam bombardment, we subjected the NRBBO: 0.11Eu^2+^ to continuous electron beam bombardment for 60 min at 6 kV and 60 mA, and tested the CL spectra. As shown in Figure S4c (Supporting Information), the CL spectra and intensity of the sample remained essentially unchanged during the 60 min of electron beam bombardment. Figure S4d (Supporting Information) shows the CL mapping of the NRBBO: 0.11Eu^2+^, from which it can be seen that NRBBO: 0.11Eu^2+^ emits light uniformly under long‐term electron beam bombardment, further indicating that NRBBO: 0.11Eu^2+^ has excellent resistance to cathodoluminescence degradation. These results confirm that NRBBO: 0.11Eu^2+^ exhibits excellent stability when operating at high energy densities, which can also be reflected in its stability under high‐energy laser excitation.

### 3.3 White Light‐Emitting Diode (WLED) Properties

**Figure 8:**
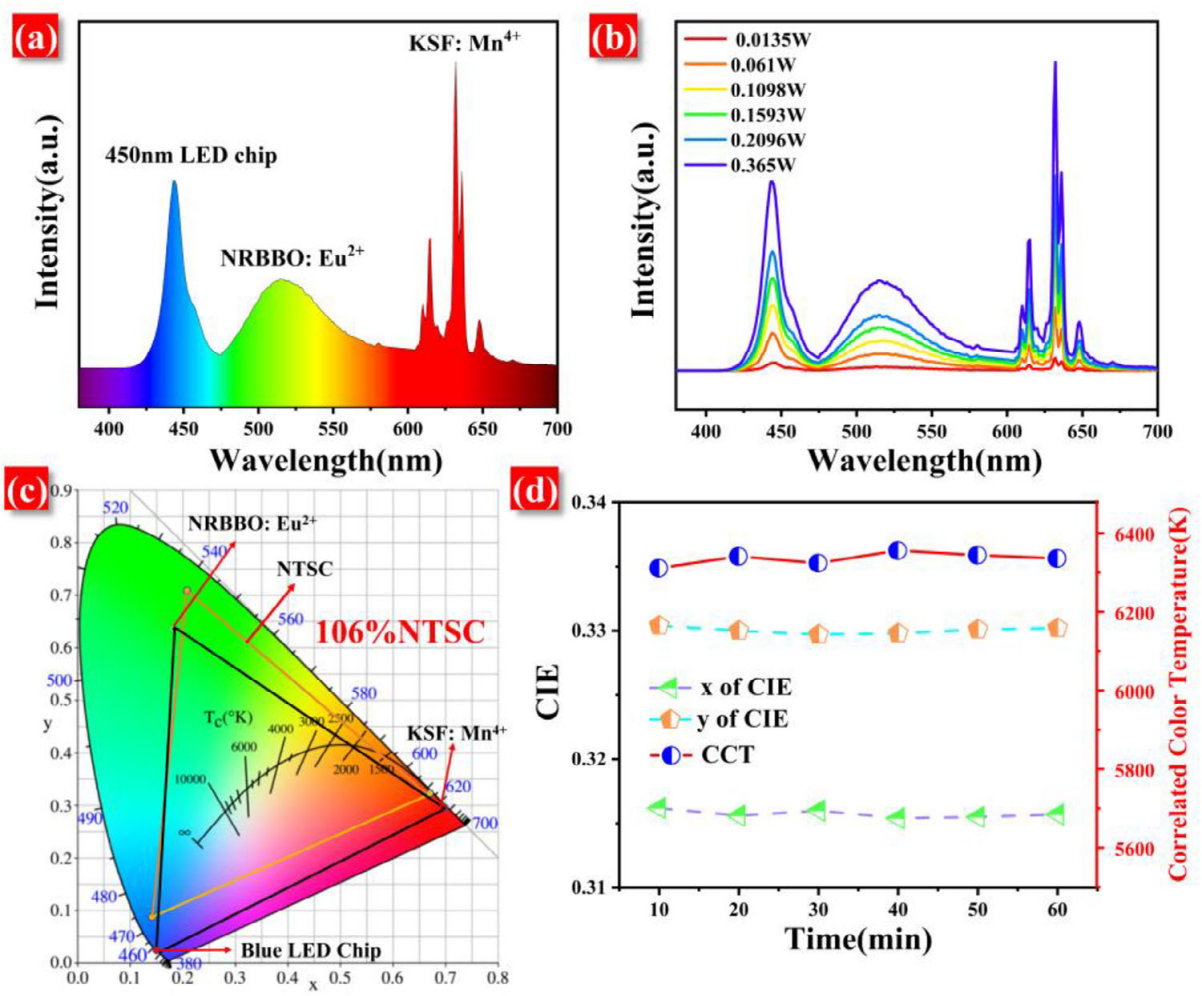
a) The emission spectrum of NRBBO: 0.11Eu^2+^ – based WLED. b) The emission spectra of NRBBO: 0.11Eu^2+^ – based WLED at different power levels. c) The color gamut of NTSC and the fabricated WLED device. d) The CIE chromaticity coordinates and CCT of the WLED devices at different power levels.

To verify the potential application of NRBBO: 0.11Eu^2+^ in backlight displays, we combined NRBBO: 0.11Eu^2+^ with KSF: Mn^4+^ and a blue GaInN chip (emission wavelength λ_em_ = 450 nm) to fabricate a WLED device. As shown in **Figure** **8**a, under a driving current of 0.1 A, the white light emitted by the LED device has a correlated color temperature (CCT) of 6336 K and CIE chromaticity coordinates of (0.315, 0.330). Figure 8b demonstrates the emission spectra of the WLED device at different power levels. With the increase of current, the intensity of the spectra is continuously enhanced while the shape and position of the spectra remain almost unchanged, indicating that the device has good stability at different power levels. Figure 8c shows that the color gamut of this WLED device is 106% of the NTSC standard. The larger color gamut enables a more realistic display of colors, and the color gamut of the device is larger than that of the devices based on the β‐SiAlON: Eu^2+^, KSF: Mn^4+^ combination blue LED chips (89%).^[^ ^52^ ^]^ Moreover, long‐term operational stability is an important indicator for assessing device performance. Excellent stability ensures that the device does not experience color drift during prolonged operation, thereby affecting the visual experience. Therefore, we tested the variation of the emission spectra of the WLED devices with time under 0.0133 W operating conditions to assess their stability. The photometric parameters in Figure 8d show that over time, the Cambridge International Education (CIE) color coordinates and CCT of the WLED device remain almost unchanged, further confirming that the WLED device we fabricated possesses outstanding stability.

## 4 Conclusion

In summary, this study demonstrates that chemical pressure induces displacement of activator ions Eu^2+^ within NBBO, enabling modulation and narrowing of the emission spectrum. By analyzing the crystal structure of NRBBO: Eu^2+^ and changes in high‐pressure spectra, we verified the presence of chemical pressure and its regulatory mechanism on the luminescent properties of NRBBO: Eu^2+^. NRBBO: 0.11Eu^2+^ exhibits excellent luminescent performance, with efficient excitation by NUV and blue light, emitting narrow‐band green light with an FWHM of 54 nm and EQE of 39%. Significantly, NRBBO: 0.11Eu^2+^ demonstrates outstanding laser excitation performance, with laser saturation threshold and luminous flux exceeding those of previously reported phosphors. Notably, the WLED device fabricated with NRBBO: 0.11Eu^2+^ achieves an NTSC color gamut of 106%. Meanwhile, the excellent CL performance demonstrates that NRBBO: 0.11Eu^2+^ exhibits stability under high energy density. These results indicate the broad application potential of NRBBO: 0.11Eu^2+^ in laser display technology. Meanwhile, the approach of using chemical pressure to modulate and narrow the FWHM of emission spectra provides new insights for developing novel narrow‐band phosphors.

## 5 Experimental Section

All samples were prepared through the following steps: First, weigh the raw materials BaCO_3_ (99.9%, Macklin), Na_2_CO_3_ (99.9%, Macklin), Rb_2_CO_3_ (99.9%, Macklin), H_3_BO_3_ (99.9%, Macklin), and Eu_2_O_3_ (99.9%, Macklin) according to the stoichiometric ratio of each element in the chemical formula. Then, grind and mix all the raw materials uniformly to obtain the mixed powder, which is transferred to an alumina crucible. Subsequently, conduct sintering in a tube furnace (N_2_/H_2_ = 95%/5%) at a temperature of 750 °C for a sintering time of 6 hours. After that, cool the samples to room temperature and grind them into powder for subsequent testing.

## Conflict of Interest

The authors declare no conflict of interest.

## Supporting information



Supporting Information

## Data Availability

The data that support the findings of this study are available from the corresponding author upon reasonable request.
