# Supplementary material for: Chemical Pressure‐Induced FWHM Narrowing in Narrowband Green Phosphors for Laser Displays with Ultra‐High Saturation Thresholds
Source: Adv Sci (Weinh). 2025 Jun 4;12(33):e05385. doi: 10.1002/advs.202505385 (PMC12412610; doi:10.1002/advs.202505385)
Supplement: Supplementary file 1 — Supporting Information [file ADVS-12-e05385-s001.docx]

**Supporting Information**

**Chemical Pressure-Induced FWHM Narrowing in Narrowband Green Phosphors for Laser Displays with Ultra-high Saturation Thresholds**

Runtian Kang, Zhezhe Su, Chunxu Bao, Yuhua Wang*

National & local Joint Engineering Laboratory for Optical Conversion Materials and Technology,

School of Materials and Energy, Lanzhou University, Lanzhou,730000, China.

* Corresponding author: Professor. Yuhua Wang, Ph. D, Professor.

E-mail: [wyh@lzu.edu.cn](mailto:wyh@lzu.edu.cn)

**Experimental**

A mixture of NRBBO: 0.11Eu^2+^ and KSF: Mn^4+^ was firstly mixed with UV curing adhesive, and then the mixed slurry was dripped onto the blue LED chip to cure to form a W-LED lamp.

**Characterization**

All samples' X-ray powder diffraction (XRD) patterns were measured by a D2-PHASER XRD diffractometer manufactured by Bruker, Germany. The X-ray source utilized Cu Kα radiation (λKα = 1.54184 Å). During the measurement process, the instrument was operated at a current of 10 mA and a voltage of 30 kV. The scanning electron microscopy (SEM) images employed in this study were obtained using an S-4800 field-emission scanning electron microscope produced by Hitachi, Japan. Energy-dispersive X-ray spectroscopy (EDS) was carried out as an accessory on the same SEM. The cathodoluminescence (CL) properties of the samples were obtained using a modified Mp-Micro-S instrument. High-resolution transmission electron microscopy (HRTEM) images and selected area electron diffraction (SAED) patterns were characterized by a TecnaiTM G2 F30 field-emission transmission electron microscope manufactured by FEI, USA, with an operating voltage of 300 kV. All spectroscopic data were acquired using an ultraviolet-visible (UV-Vis) fluorescence spectrometer produced by Horiba Jobin Yvon, France. The quantum efficiency (QE) was measured by using the Hamamatsu C9920-02,-03 Absolute PL Quantum Yield Measurement System.

The calculations were obtained using the Cambridge serial total energy package (CASTEP) module in Materials Studio. The calculations were performed using Perdew-Burke-Ernzerhof (PBE) functional based on the generalized gradient approximation (GGA). Atomic positions are relaxed using the Broyden-Fletcher-Goldfarb-Shanno (BFGS) method. The structural optimization parameters are as follows: energy set to 1.0e^-5^ eV/Å, maximum force set to 0.03 Ha/Å, and maximum displacement set to 0.001 Å, respectively.


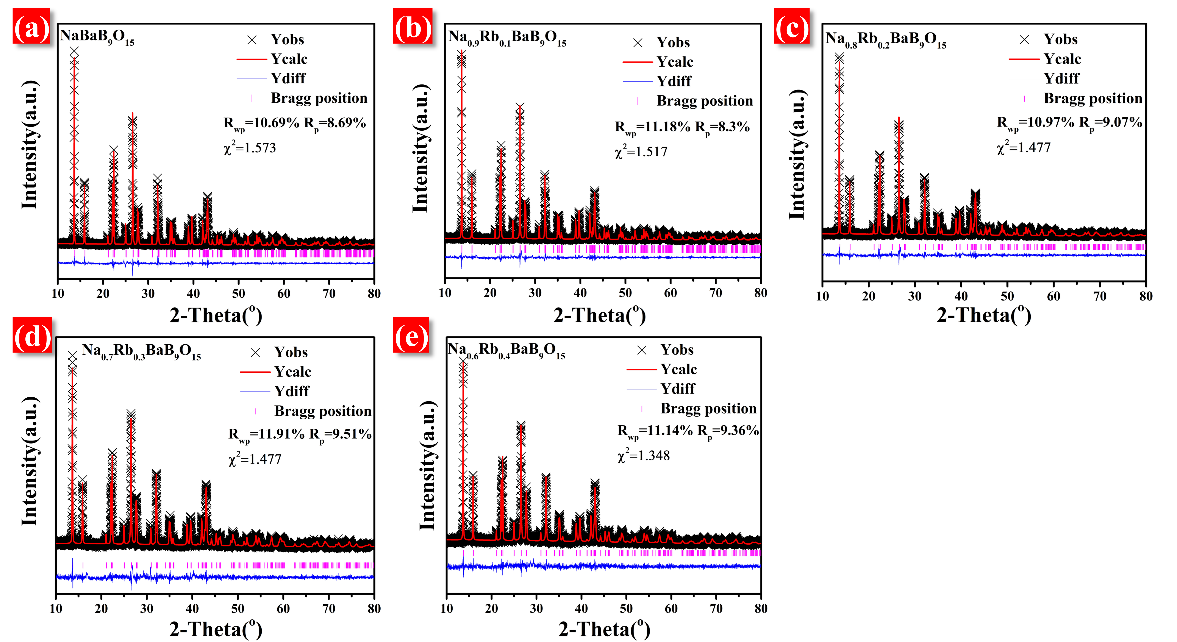


Figure. S1 XRD refinement of N_1-x_R_x_BBO (0 ≤ x ≤ 0.4).

Table S1 Crystallographic data of NaBaB_9_O_15_ by the Rietveld refinement

|  | NaRbBaB_9_O_15_ |
| --- | --- |
| Space group | *R*3*c* |
| Crystal system | trigonal |
| Cell parameters (Å) | a = b = 11.0706(12)  c = 17.3549(19) |
| Cell ratio | c/a= 1.5677 |
| Z | 6 |
| Cell volume (Å^3^) | 1842.02(50) |
| Reliability factors | χ^2^=1.573, R_p_=8.7%, R_wp_=10.7% |

Table S2 Crystallographic data of Na_0.9_Rb_0.1_BaB_9_O_15_ by the Rietveld refinement

|  | Na_0.9_Rb_0.1_BaB_9_O_15_ |
| --- | --- |
| Space group | *R*3*c* |
| Crystal system | trigonal |
| Cell parameters (Å) | a = b = 11.0756(12)  c = 17.3517(19) |
| Cell ratio | c/a= 1.5667 |
| Z | 6 |
| Cell volume (Å^3^) | 1843.4(50) |
| Reliability factors | χ^2^=1.517, R_p_=8.3%, R_wp_=11.18% |

Table S3 Crystallographic data of Na_0.8_Rb_0.2_BaB_9_O_15_ by the Rietveld refinement

|  | Na_0.8_Rb_0.2_BaB_9_O_15_ |
| --- | --- |
| Space group | *R*3*c* |
| Crystal system | trigonal |
| Cell parameters (Å) | a = b = 11.0804(2)  c = 17.3467(4) |
| Cell ratio | c/a= 1.5655 |
| Z | 6 |
| Cell volume (Å^3^) | 1844.40(6) |
| Reliability factors | χ^2^=1.477, R_p_=9.07%, R_wp_=10.97% |

Table S4 Crystallographic data of Na_0.7_Rb_0.3_BaB_9_O_15_ by the Rietveld refinement

|  | Na_0.7_Rb_0.3_BaB_9_O_15_ |
| --- | --- |
| Space group | *R*3*c* |
| Crystal system | trigonal |
| Cell parameters (Å) | a = b = 11.0906(2)  c = 17.3321(5) |
| Cell ratio | c/a= 1.5628 |
| Z | 6 |
| Cell volume (Å^3^) | 1846.27(8) |
| Reliability factors | χ^2^=1.477, R_p_=9.51%, R_wp_=11.91% |


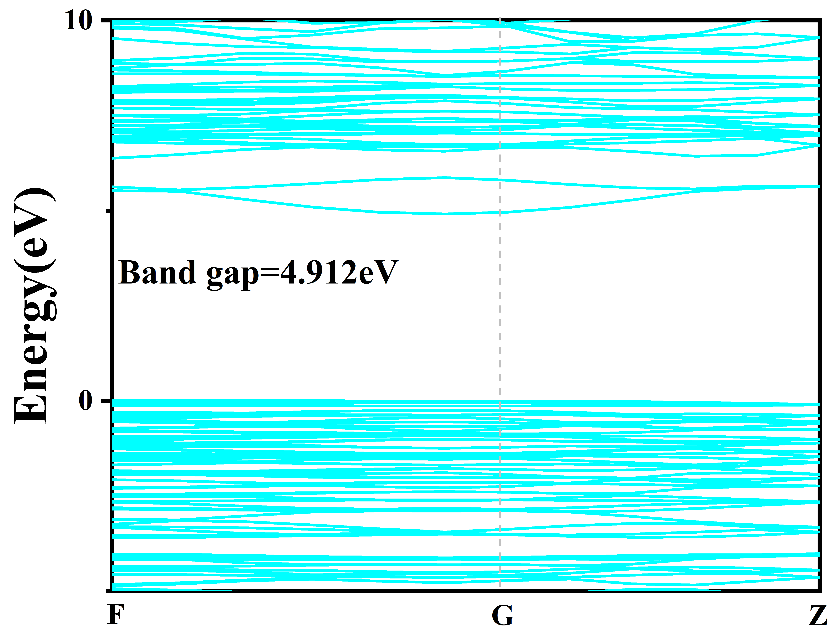


Figure. S2 Band structure of NRBBO.


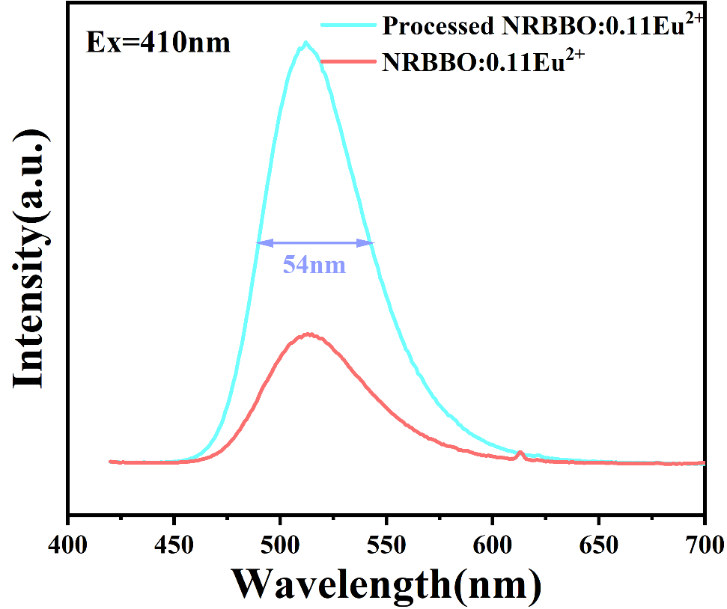


Figure. S3 The emission spectra of the N_0.6_R_0.4_BBO: 0.11Eu^2+^ with and without heat treatment.

Calculation 1:

$$\begin{aligned} D_{r}=100\times\frac{\left[ R_{m}\left( \mathrm{CN} \right)-\left[ R_{d}\left( \mathrm{CN} \right) \right] \right]}{R_{m}\left( \mathrm{CN} \right)}\#1 \end{aligned}$$

Where R_m_(CN) and R_d_(CN) represent the radii of the substituted and doped ions, respectively, in the same coordination number(CN).

Calculation 2:

$$\begin{aligned} E=Q\left[ 1-\left( \frac{V}{4} \right)^{\frac{1}{V}} \right]{10}^{-\frac{nE_{a}r}{80}}\#2 \end{aligned}$$

Where Q is the energy position of the lower edge of the d-band of the free ion of Eu^2+^, which is 34,000 cm-1, V is the valence state of the rare-earth ion, n is the number of anions near the shell around Eu^2+^, Ea is the electron affinity of the anion, and r is the radius of the host cation that has been replaced by Eu^2+^.

Calculation 3:

$$\begin{aligned} E_{f}=\frac{E_{d}-\left( E_{P}-n\mu_{Y}+n\mu_{X} \right)}{n}\#3 \end{aligned}$$

The formation energy is defined as the energy required to insert n atoms of X after removing n atoms of Y. In this formula, E_f_ represents the formation energy per defect, E_d_ represents the energy of the doped system, E_p_ represents the energy of the undoped system, n represents the number of doped atoms, μ_X_ represents the chemical potential of the doped atoms, and μ_Y_ represents the chemical potential of the atoms being replaced in the original system.

The computational results are obtained by applying the first principles approach with the help of the Cambridge Serialized Total Energy Package (CASTEP) module in the Materials Studio software. The specific computational procedure was carried out using Density Functional Theory (DFT) and is based on the Perdew-Burke-Ernzerhof (PBE) generalization under the Generalized Gradient Approximation (GGA) in conjunction with the Projected Augmented Wave (PAW) method.

The specific parameter settings regarding the optimization of the structures were as follows: the threshold for the energy was set to 1.0 × 10^-5^ eV/Å, the maximum force was limited to 0.03 Ha/Å and the maximum displacement was set to 0.001 Å. The maximum displacement was set to 0.001 Å, and the maximum force was set to 0.001 Ha/Å.

Calculation 4:

$$\begin{aligned} FWHM=\sqrt{8ln2}ћ\omega\sqrt{S\cot h\left( \frac{ћ\omega}{2KT} \right)\mathrm{KT}}\#4 \end{aligned}$$

Where k is the Boltzmann constant, and S and ℏω denote respectively the Huang-Rhys factor and the quantized minimum phonon energy. The relationship can be obtained by approximation and conversion as follows:

$$\begin{aligned} \left( FWHM \right)^{2}=a+b*\left( 2KT \right)\#5 \end{aligned}$$

where a= 5.57 * S * (ℏ𝜔)^2^, b=5.57 * S * (ℏ𝜔).


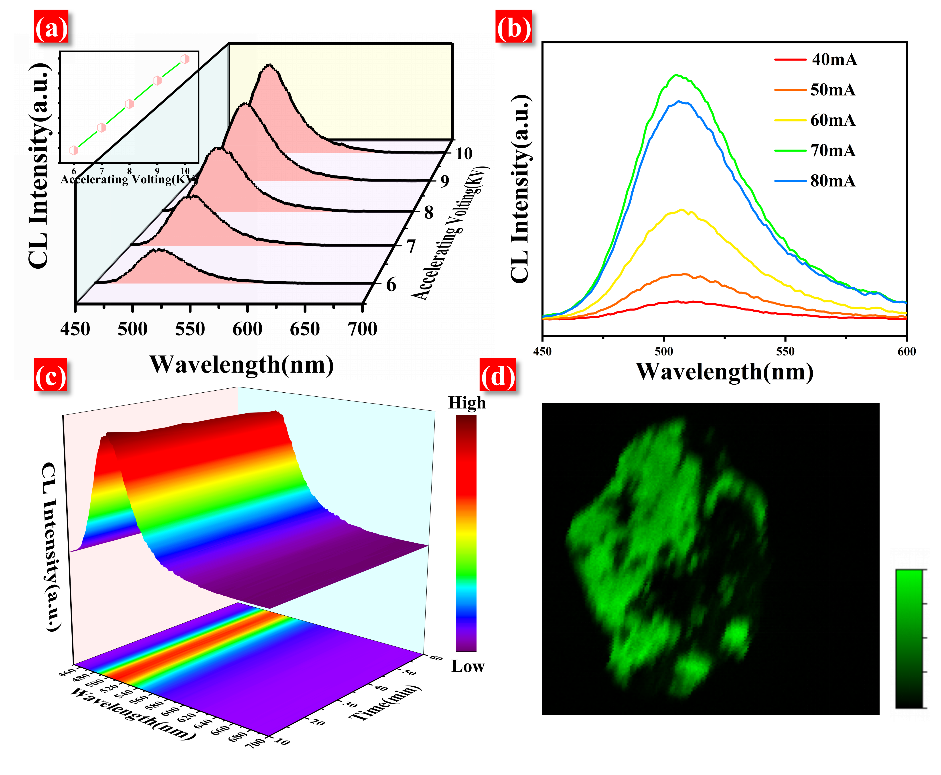


Figure. S4 CL spectra of NRBBO: 0.11Eu^2+^ with different (a) accelerating voltages and (b) probe currents. (c) Anti-degradation properties of the NRBBO: 0.11Eu^2+^. (d) CL mapping of NRBBO: 0.11Eu^2+^

Calculation 5

$$\begin{aligned} L=250\left( \frac{A}{\rho} \right)\left( \frac{E}{\sqrt{Z}} \right)^{n}，n=\frac{1.2}{1-0.29logZ}\#\#6 \end{aligned}$$

Where Z, e, ρ, and A represent the number of electrons in a molecule, the accelerating voltage (kV), the density of phosphor, and the relative molecular mass, respectively. For this system, Z = 248, ρ = 2.81g/cm^3^, A = 522.59g/mol.
